# SELF-GLUCOSE MONITORING AND HYPOGLYCEMIA RISK IN OLDER RURAL VETERANS WITH TYPE 2 DIABETES

**DOI:** 10.1093/geroni/igz038.980

**Published:** 2019-11-08

**Authors:** Willy Marcos Valencia, Kaicheng Wang, Kiranmayee Muralidhar, Stuti Dang

**Affiliations:** 1 Miami Veterans Affairs Healthcare System- GRECC, Miami, Florida, United States; 2 Department of Public Health Sciences, University of Miami Miller School of Medicine, Miami, Florida, United States; 3 Miami VA GRECC, Miami, Florida, United States

## Abstract

Hypoglycemia evaluation is expected in every encounter with diabetic patients. However, self-monitoring and self-management may not be complete at home, and limited by geriatric syndromes. Furthermore, hypoglycemia risk increases with age, and rurality may limit access to frequent monitoring. We identified 112 rural veterans with high hypoglycemia risk, using the local medication database (sulfonylureas and insulin), combined with age and glycated hemoglobin (HbA1c). Statistical analyses were conduct using SAS 9.4 (Cary, NC). We used Chi-square, Fisher’s, One-way ANOVA for baseline variables, and a multivariate logistic regression model to assess the association of hypoglycemia and risk factors, including age, HbA1c%, self-monitoring, and knowledge. Hypoglycemia was reported in 30.4% of cases, of whom the majority were younger than those not reporting hypoglycemia (72.0±4.3 vs 75.0±6.5 years, p=.015). Baseline HbA1c% was higher in cases with hypoglycemia compared to those without (7.7±1.6% versus 7.3±1.2%, not statistically significant). There were no significant differences between pharmacologic regimens, self-monitoring, and general knowledge. Veterans who knew hypoglycemia symptoms were 6 times more likely to reported hypoglycemia, compared with veterans who did not know any symptoms. We contacted primary care teams (PCT) for whom medications were adjusted. Hypoglycemia risk is high in the older population, and telemedicine programs can support primary care teams to improve management of their patients. Poor symptom knowledge needs to be addressed, while considering special attention for hypoglycemia unawareness in the oldest age group. We are implementing a project using continuous glucose monitoring in this high-risk population.

